# Characteristics of Resting-State Functional Connectivity in Intractable Unilateral Temporal Lobe Epilepsy Patients with Impaired Executive Control Function

**DOI:** 10.3389/fnhum.2017.00609

**Published:** 2017-12-13

**Authors:** Chao Zhang, Hongyu Yang, Wen Qin, Chang Liu, Zhigang Qi, Nan Chen, Kuncheng Li

**Affiliations:** ^1^Department of Radiology, Xuanwu Hospital, Capital Medical University, Beijing, China; ^2^Beijing Key Laboratory of Magnetic Resonance Imaging and Brain Informatics, Capital Medical University, Beijing, China; ^3^Department of Radiology and Tianjin Key Laboratory of Functional Imaging, Tianjin Medical University General Hospital, Tianjin, China; ^4^Department of Functional Neurosurgery, Xuanwu Hospital, Capital Medical University, Beijing, China

**Keywords:** executive control function, functional connectivity, resting-state networks, temporal lobe epilepsy, Wisconsin Card Sorting Test, independent component analysis

## Abstract

Executive control function (ECF) deficit is a common complication of temporal lobe epilepsy (TLE). Characteristics of brain network connectivity in TLE with ECF dysfunction are still unknown. The aim of this study was to investigate resting-state functional connectivity (FC) changes in patients with unilateral intractable TLE with impaired ECF. Forty right-handed patients with left TLE confirmed by comprehensive preoperative evaluation and postoperative pathological findings were enrolled. The patients were divided into normal ECF (G1) and decreased ECF (G2) groups according to whether they showed ECF impairment on the Wisconsin Card Sorting Test (WCST). Twenty-three healthy volunteers were recruited as the healthy control (HC) group. All subjects underwent resting-state functional magnetic resonance imaging (rs-fMRI). Group-information-guided independent component analysis (GIG-ICA) was performed to estimate resting-state networks (RSNs) for all subjects. General linear model (GLM) was employed to analyze intra-network FC (*p* < 0.05, false discovery rate, FDR correction) and inter-network FC (*p* < 0.05, Bonferroni correction) of RSN among three groups. Pearson correlations between FC and neuropsychological tests were also determined through partial correlation analysis (*p* < 0.05). Eleven meaningful RSNs were identified from 40 left TLE and 23 HC subjects. Comparison of intra-network FC of all 11 meaningful RSNs did not reveal significant difference among the three groups (*p* > 0.05, FDR correction). For inter-network analysis, G2 exhibited decreased FC between the executive control network (ECN) and default-mode network (DMN) when compared with G1 (*p* = 0.000, Bonferroni correction) and HC (*p* = 0.000, Bonferroni correction). G1 showed no significant difference of FC between ECN and DMN when compared with HC. Furthermore, FC between ECN and DMN had significant negative correlation with perseverative responses (RP), response errors (RE) and perseverative errors (RPE) and had significant positive correlation categories completed (CC) in both G1 and G2 (*p* < 0.05). No significant difference of Montreal Cognitive Assessment (MoCA) was found between G1 and G2, while intelligence quotient (IQ) testing showed significant difference between G1and G2.There was no correlation between FC and either MoCA or IQ performance. Our findings suggest that ECF impairment in unilateral TLE is not confined to the diseased temporal lobe. Decreased FC between DMN and ECN may be an important characteristic of RSN in intractable unilateral TLE.

## Introduction

Cognitive impairment is a common complication of epilepsy that can decrease quality of life and increase condition-associated costs (Bell et al., 2011; Keezer et al., 2016). Executive control function (ECF) has been increasingly reported as a component separate from memory, language and other cognitive abilities traditionally used for evaluating cognitive function (Royall et al., 2002). ECF deficits can lead to complex clinical symptoms in temporal lobe epilepsy (TLE) patients, with devastating effects on the patient’s ability to maintain activities of daily life and possibly increasing the risk of suicide (Lin et al., 2012; Keezer et al., 2016). Early detection of ECF impairment would be useful for prompting intervention and delaying or halting the progression of functional damage (Grafman and Litvan, 1999; Lin et al., 2012; Fazel et al., 2013).

The pathogenetic mechanism of TLE with ECF impairment remains unclear (Royall et al., 2002). Both morphological and diffusion tensor imaging (DTI) researches on ECF in TLE patients had conflicting results. Such as, early studies reported that the hippocampus may be a major contributor to ECF in TLE (Corcoran and Upton, 1993; Giovagnoli, 2001); however, another study believed that prefrontal cortex (PFC) was a key structure (Keller et al., 2009). Similarly, this contradiction was also found in DTI studies (Wang et al., 2007; Kucukboyaci et al., 2012; Widjaja et al., 2013). According to recent reports, the most likely explanation of ECF was that the critical determinants of ECF impairment in patients with TLE were most related to frontal lobe system abnormalities and damage to pathways linking related brain structures rather than associating with a single structure dysfunction (Martin et al., 2000; Kim et al., 2007; Tuchscherer et al., 2010; Widjaja et al., 2013). But it is still awaited to be elucidated.

A growing number of resting-state functional magnetic resonance imaging (rs-fMRI) studies were performed to assess resting-state networks (RSNs) in TLE patients with cognitive impairment. Cognitive function alterations in MTLE could be reflected by correlated RSN abnormalities through intra-network functional connectivity (FC) analysis, including impaired perceptual function and working memory (Zhang et al., 2009; Ibrahim et al., 2014). The result may help us understanding the pathophysiological mechanisms underlying cognitive impairments in mTLE. In addition, Vollmar et al. (2011) found the underlying mechanism of myoclonic jerks in juvenile myoclonic epilepsy was correlated with multiple structures based on functional integration analysis of RSNs. FMRI studies provided us new insight in studying the mechanism of epilepsy. To our knowledge, no study has investigated FC alteration within and between RSNs in patients with ECF impairment.

The Wisconsin Card Sorting Test (WCST) is recognized as a “gold standard” for ECF testing and is probably the most effective measurement tool (Royall et al., 2002). The WCST consists of 13 test items to comprehensively evaluate ECF. Therefore, WCST was employed as a standard for judging ECF performance and ECF testing results by WCST was used as the grouping standard in our study (Martin et al., 2000; Royall et al., 2002; Kim et al., 2007). Based on this standard, we investigated resting-state FC changes in unilateral intractable TLE patients with and without impaired ECF.

Rs-fMRI can detect FC changes between RSNs by correlating fluctuations in blood oxygen level-dependent (BOLD) signal (Biswal et al., 1995; Power et al., 2014). Previous studies reported that resting-state FC was not only able to reflect the brain network features but also provided a promising tool for exploring pathophysiological mechanism of disease (Stam et al., 2009; Baggio et al., 2015a; Liu et al., 2015; Wu et al., 2015), furthermore, FC changes was demonstrated closely relating to cognitive impairment (Baggio et al., 2015b; Browndyke et al., 2017; Pereira et al., 2017). Importantly, understanding interactions among the whole brain network is considered to be more valuable than studying a single RSN when exploring cognitive mechanisms (Petersen and Sporns, 2015). Group-information-guided independent component analysis (GIG-ICA) can identify subject-specific independent components (ICs) across different groups with stronger independence and better spatial correspondence, making it possible to compare FC between large-scale networks among different groups (Du and Fan, 2013; Du et al., 2015). On the other hand, RSNs were demonstrated to be associated with regional blood supply and electroencephalography (EEG) patterns (Seeley et al., 2007; Liang et al., 2013; Ma et al., 2016). In the present study, we employed GIG-ICA to investigate FC alterations within and between RSNs in TLE patients with and without ECF impairment to observe the characteristics of brain network connectivity in TLE patients with ECF dysfunction and to further explore the pathogenesis of this condition.

## Materials and Methods

The study protocol was approved by the Ethics Committee of Xuanwu Hospital, Capital Medical University, Beijing, China. Written informed consent was obtained from each participant in accordance with the Declaration of Helsinki.

Forty patients with left intractable TLE proved by comprehensive preoperative evaluation and postoperative pathological findings were enrolled (Table 1). All the patients underwent a comprehensive assessment including neuropsychological and clinical symptoms and findings from MRI, magnetoencephalography, EEG and stereo-EEG examinations. ECF performances were evaluated based on WCST results (Kim et al., 2007), including response errors (RE), perseverative responses (RP), perseverative errors (RPE), nonperseverative errors (NRPE) and categories completed (CC). Normal WCST scores for all five parameters indicated good ECF, those with scores outside acceptable ranges were considered to have ECF impairment. The acceptable range was defined based on the T-score of RP through all subjects, and the final threshold of original RP judging ECF at normal level was less than 30 (Kim et al., 2007). These findings were used to divide patients into two subgroups: normal ECF was designated as subgroup 1 (G1: 18, 12 male and 6 female; mean age: 25.3 years; range: 16–38 years; mean education: 12.6 years; range: 6–19 years) and patients with impaired ECF were in subgroup 2 (G2: 22, 10 male and 12 female; mean age: 25.7 years; range: 13–35 years; mean education: 11.5 years; range: 6–19 years). All the patients underwent routine medication, none of them have received any other relevant interventions, no significant difference of age at seizure onset and seizure duration between G1 and G2 group (Table 1). None of the patients had extra-temporal lobe brain lesions. Thirty-one patients were pathologically identified as having hippocampal sclerosis (HS). The other nine patients had neocortical TLE confirmed by postoperative pathological studies. Twenty-three healthy volunteers (13 male and 10 female; mean age: 26.7 years; range: 23–35 years; mean education: 12.9 years; range: 6–19 years) were recruited as a healthy control (HC) group and completed the same neuropsychological tests. There was no significant group difference in age, sex, or education between the HC and patient groups (Table 1).

**Table 1:** Group demographics and clinical information of all subjects.

Variable	G1 (*N* = 18)	G2 (*N* = 22)	HC (*N* = 23)	Between-group comparisons (*p* values)		
				G1 vs. G2	G1 vs. HC	G2 vs. HC
Gender (M/F)	12/6	12/10	13/10		0.725*	
Age (years)	16–38	13–35	23–35		0.695*	
	25.3 ± 6.9	25.7 ± 5.9	26.7 ± 3.0			
Education (years)	6–19	6–19	6–19		0.611*	
	12.6 ± 3.9	11.5 ± 4.1	12.9 ± 3.9			
MoCA	18–27	15–28	19–28	0.088	0.068	0.000
	23.2 ± 2.3	21.5 ± 2.7	24.8 ± 2.0			
IQ	81–110	72–103	85–111	0.047	1.000	0.002
	93.4 ± 2.3	87.1 ± 9.2	95.5 ± 6.4			
Age at seizure onset	5–30	3–24	N/A	0.326	N/A	N/A
	12.4 ± 7.1	10.4 ± 6.2	N/A			
Duration of TLE	4–23	3–30	N/A	0.300	N/A	N/A
	13.1 ± 6.1	15.2 ± 6.8	N/A			

M, male; F, female; Edu, education; MoCA, Montreal Cognitive Assessment; IQ, intelligence quotient; G1, subgroup 1; G2, subgroup 2; HC, healthy controls; N/A, not applicable. Data are presented as range and mean ± SD. *The value represent the comparison between three groups.

To avoid the influence of confounding factors that may also affect executive function, the Montreal Cognitive Assessment (MoCA) for cognitive performance and Wechsler Adult Intelligence Scale (WAIS) to measure the intelligence quotient (IQ) were also administered.

### MRI Data Acquisition

All participants were scanned on a 3.0T Magneton Trio Tim MRI scanner (Siemens Healthcare, Erlangen, Germany) with a 32-channel phase-array head coil. Conventional brain axial fluid-attenuated inverse recovery (FLAIR) sequence scanning was performed to exclude other brain abnormalities before rs-fMRI. Tight but comfortable foam padding was used to minimize head motion, and the subjects wore earplugs to reduce imaging noise. The participants were asked to stay calm, close their eyes, breath smoothly and avoid thinking of anything specific. Resting BOLD images were acquired using an echo-planar imaging sequence with the following parameters: time of repetition/time of echo = 2000/30 ms, field of view = 220 mm × 220 mm, GRAPPA (PE; GeneRalized Autocalibrating Partial Parallel Acquisition phase encoding) 2, slice thickness = 3 mm, voxel size = 3.4 mm × 3.4 mm × 3.0 mm, 35 slices, flip angle = 90°, and total acquisition time = 6:08 min and 180 volumes. T1-weighted images were also acquired using a 3D-MP-RAGE (three-dimensional three-dimension magnetization-prepared rapid gradient-echo) providing isotropic voxels of 1 mm × 1 mm × 1 mm.

### Data Preprocessing

The rs-fMRI data for all subjects were preprocessed using data Processing and Analysis for (Resting-State) Brain Imaging (DPABI; Yan et al., 2016). The first 10 volumes of each functional time series were removed since the signals had not yet reached equilibrium. Slice timing was performed to correct differences between all slices, and the other volumes were then realigned with the first. The Friston-24 model was used for individual-level head motion correction for all subjects, and any subject who had a maximum displacement >2 mm, a maximum rotation >2.0°, or mean framewise displacement (FD) >0.3 was excluded from the group. In addition, we set mean FD as a covariate for the group-level statistics of our study to minimize the impact of potential head movements (Yan et al., 2013). The fMRI data were then normalized to the standard echo planar imaging template and resampled to 3-mm cubic voxels. Finally, fMRI data were smoothed (6-mm full width at half maximum). As described in previous studies, we did not choose global signal regression for our data (Scholvinck et al., 2010; Chai et al., 2012; Saad et al., 2012; Yang et al., 2014).

### Independent Component Analysis

After data preprocessing, ICs for all subjects were analyzed through GIG-ICA using GIFT software (version 2.0a)^1^. Three steps for fMRI data decomposition were based on this toolbox: (i) data reduction; (ii) applying the ICA algorithm; and (iii) back-reconstruction for individual-level components. Finally, 24 ICs were auto-estimated though MELODIC criteria and 100 times with GICA. Eleven meaningful ICs were identified as functionally related RSNs via visual inspection and previous reports related to RSNs. The subject-specific spatial maps were transformed to z scores.

### Statistical Analysis of Neuropsychological Tests

To eliminate the potential impact of other variables as much as possible, we employed a general linear model (GLM) analysis of neuropsychological tests with age, sex and education as covariates to identify significant differences between the HC and patient groups (Table 1). Pearson correlations between FC, MoCA and IQ tests were also calculated though partial correlation analysis with age, sex and education as covariates (*p* < 0.05).

### Functional Network Connectivity Analysis

First, we removed head motion parameters, BOLD signals of the ventricles, white matter, and the whole brain as nuisance covariates from the datasets. Then, a random-effect one-sample *t*-test was performed on each meaningful IC in statistical parametric mapping (SPM) 8 using a false discovery rate (FDR) correction (*p* < 0.05 and *T* = 8) and cluster size of 100 voxels to create a sample-specific component map (Supplementary Figure S1; Song et al., 2013; Wang et al., 2014).

For each individual, the mean time course of each RSN was obtained by averaging the time course of all voxels within each sample-specific RSN mask. Pearson correlation coefficients of the mean time courses between each pair of RSNs of individuals were calculated and then converted to z values though Fisher’s r-to-z transformation to improve normality. The next step, a random effect one-sample *t*-test was used to test individuals’ z values to determine whether a correlation between each pair of RSNs of each group was statistically significant (*p* < 0.05). Intergroup comparisons were only performed on the condition that inter-network FC reached statistical significance in each group. A GLM with age, sex, education, IQ and mean FD as covariates was used to analyze which pairs of inter-network FC components were significantly different between G1, G2 and HC, and *post hoc* contrasts were tested to observe inter-network FC differences between each pair of groups (*p* < 0.05, Bonferroni correction). Pearson correlations between FC and each WCST parameter were calculated though partial correlation with age, sex, education, IQ and mean FD as covariates (*p* < 0.05). Statistical tests were carried out in SPSS version 16 (SPSS Inc., Chicago, IL, USA).

### Intra-Network FC of RSNs Analysis

Intra-network FC differences of all RSNs extracted in this study were also tested using a GLM analysis with age, sex, education, IQ and mean FD as covariates in a voxel-wise manner by SPM. Multiple comparisons were corrected using family-wise error correction (FDR correction; *p* < 0.05) through SPM.

## Results

### RSNs

A total of 24 components were extracted by GIG-ICA. The 11 meaningful RSNs across all subjects were the dorsal sensorimotor-related network (dSMN), executive control network (ECN), occipital pole visual network (pVN), medial VN (mVN), anterior default-mode network (aDMN), left frontoparietal network (lFPN), right FPN (rFPN), visuospatial network (VSN), ventral SMN (vSMN), DMN and posterior part of DMN (pDMN), that all of the RSNs were consistent with previous reports (Supplementary Figure S1; Mantini et al., 2007; Seeley et al., 2007; Yeo et al., 2011; Wang et al., 2014; Du et al., 2015; Raichle, 2015; de Campos et al., 2016; Ma et al., 2016).

### FC Changes

Comparison of intra-network FC of all 11 meaningful RSNs showed no significant FC difference in each RSNs among the three groups (FDR correction, *p* > 0.05).

**Figure 1:**
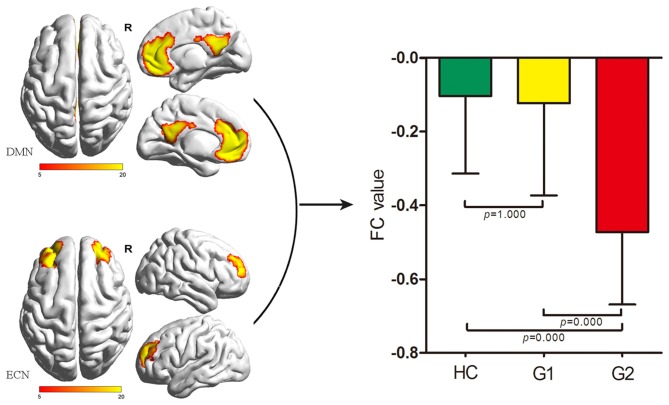
Comparisons of inter-network FC alterations between the ECN and DMN among HC, G1 and G2. G2 exhibited decreased FC between the ECN and DMN compared with G1 and HC (both *p* = 0.000, Bonferroni corrected); G1 showed no significant difference of FC between the ECN and DMN compared with HC. DMN, default-mode network; ECN, executive control network; FC, functional connectivity; HC, healthy control; G1, subgroup 1; G2, subgroup 2.

As for inter-network comparison, we only found a significant FC difference between the DMN and ECN (*p* = 0.000) among G1, G2 and HC. G2 showed significantly decreased FC between the DMN and ECN when compared with G1 and HC (Bonferroni corrected, both *p* < 0.05). There was no significant difference between G1 and HC (*p* = 1.000; Figure 1). There was no significant difference of FC between any other RSN pair extracted in the present study.

### Neuropsychological Test Score Correlations

The mean MoCA score of HC was 24.8 ± 2.0 and the range was 19–28; and the score of G1 and G2 was 23.2 ± 2.3 and 21.5 ± 2.7, the range was 18–27 and 15–28, respectively.

**Figure 2:**
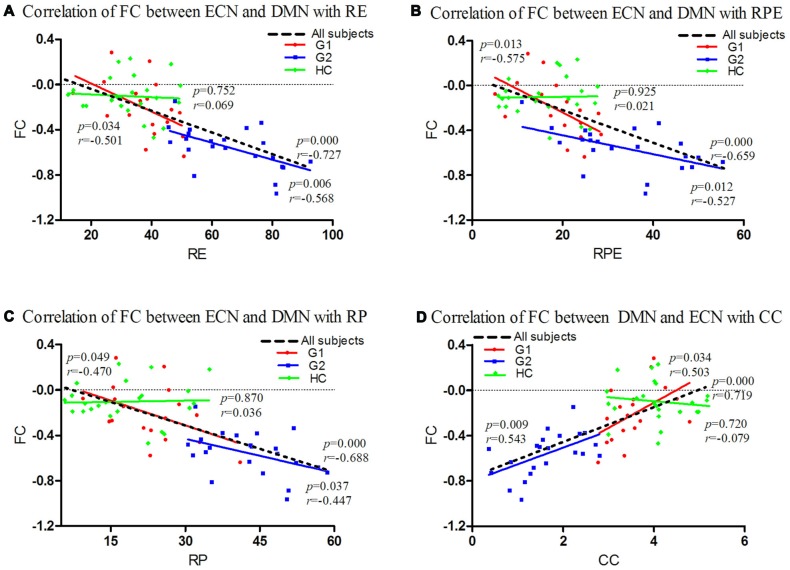
Correlations of FC between ECN and DMN with Wisconsin Card Sorting Test (WCST) scores in all subjects. FC between the ECN and DMN was significantly negatively correlated with perseverative RE (G1: *p* = 0.034, *r* = −0.501; G2: *p* = 0.006, *r* = −0.568; all subjects: *p* = 0.000, *r* = −0.727; **A**), RPE (G1: *p* = 0.013, *r* = −0.575; G2: *p* = 0.012, *r* = −0.527; all subjects: *p* = 0.000, *r* = −0.659; **B**), RP (G1: *p* = 0.049, *r* = −0.470; G2: *p* = 0.037, *r* = −0.447; all subjects: *p* = 0.000, *r* = −0.688; **C**) and had significant positive correlation with CC (G1: *p* = 0.034, *r* = 0.503; G2: *p* = 0.009, *r* = 0.543; all subjects: *p* = 0.000, *r* = 0.719; **D**). For HC analysis, no significant correlation was found between FC with WCST scores (*p* > 0.05; **A–D**). CC, categories completed; DMN, default-mode network; ECN, executive control network; FC, functional connectivity; HC, healthy controls; G1, subgroup 1; G2, subgroup 2; RE, response errors; RP, perseverative responses; RPE, perseverative errors.

A GLM analysis for MoCA and IQ with age, sex, and education as covariates showed significant differences in MoCA scores between G2 and HC, however, there was no significant difference between G1 and HC or G1 and G2 (Table 1). For IQ analysis, significant differences were found between G2 and HC as well as between G1 and G2, and there was no significant difference between G1 and HC (Table 1). Additionally, we did not find any significant correlation between FC and MoCA (*p* = 0.444, *r* = −0.214 and *p* = 0.974, *r* = 0.008) or IQ (*p* = 0.401, *r* = 0.234 and *p* = 0.156, *r* = −0.339) when comparing G1 and G2 (Figure 2). Significant difference of IQ may effect on the ECF performance (Sherman et al., 2014). Therefore, IQ effect was included as a covariate in statistical analysis of FC.

For the correlation analysis between ECN/DMN FC and neuropsychological test score, we found that FC between the ECN and DMN was significantly negatively correlated with perseverative RE (G1: *p* = 0.034, *r* = −0.501; G2: *p* = 0.006, *r* = −0.568; all subjects: *p* = 0.000, *r* = −0.727), RPE (G1: *p* = 0.013, *r* = −0.575; G2: *p* = 0.012, *r* = −0.527; all subjects: *p* = 0.000, *r* = −0.659), RP (G1: *p* = 0.049, *r* = −0.470; G2: *p* = 0.037, *r* = −0.447; all subjects: *p* = 0.000, *r* = −0.688) and had significant positive correlation with CC (G1: *p* = 0.034, *r* = 0.503; G2: *p* = 0.009, *r* = 0.543; all subjects: *p* = 0.000, *r* = 0.719). For HC analysis, no significant correlation was found between FC with WCST scores (*p* > 0.05; Figure 2).

## Discussion

In this study, we found significantly decreased FC between the ECN and DMN in ECF-impaired TLE patients compared with TLE patients without ECF impairment and HCs. Furthermore, we found that FC between the ECN and DMN is significantly positively correlated with ECF. Although MoCA and IQ performance were different between patients with and without ECF impairment, no correlation was detected between FC and these test scores in either subgroup.

### TLE with ECF Impairment

Abnormal FC between the ECN and DMN in TLE patients with ECF impairment is consistent with the point of view that it is related to brain network dysfunction (Bernhardt et al., 2011; DeSalvo et al., 2014; Vaughan et al., 2016). Our results were also similar to the hypothesis of previous structural studies that multiple brain regions were involved in TLE patients with cognitive impairment (Kim et al., 2007; Tuchscherer et al., 2010; Widjaja et al., 2013; Leyden et al., 2015). There was still lack of elaboration focusing on the relationship between abnormal structures associating with ECF impairment in previous researches. Furthermore, ECF dysfunction related networks had not been determined yet in TLE patients. In this study, we investigated FC alterations in RSNs between subgroups of TLE patients with and without ECF impairment, and we also evaluated the relationship between FC and ECF performance. We found no significant intra-network FC difference between patients with and without ECF impairment, the inter-network analysis revealed diminished FC between the ECN and DMN in patients with poor ECF performance. This may indicate that functional integration of the whole brain network may be more sensitive than functional changes in a single RSN in detecting ECF impairment from the full TLE patient group in this study (Petersen and Sporns, 2015), and abnormal FC between ECN and DMN may reflect the brain network characteristics of ECF impairment in TLE.

Our study identified two core areas of the DMN and ECN associated with ECF. The DMN is the most well-studied RSN and covers the precuneus/posterior cingulate cortex (PCC); medial PFC (MPFC); and medial, lateral, and inferior parietal cortex (IPC; Mantini et al., 2007; Brewer et al., 2011; Mohan et al., 2016). The DMN is important for cognitive performance including language ability (Oser et al., 2014), episodic memory capacity (McCormick et al., 2014) and attentional control (Jiang et al., 2017). A few studies have reported functional organization abnormalities of the DMN in TLE patients with ECF impairment (Bossong et al., 2013; Crittenden et al., 2015). The ECN is accepted as being closely involved in cognitive domains including memory, emotion, and behavior (Seeley et al., 2007; Smith et al., 2009). The DLPFC, as the core area of ECN, was considered to be more vital for ECF (Miller and Cohen, 2001). And, ECF impairment was demonstrated associating with ECN abnormal in different types of imaging studies (Takaya et al., 2006; Keller et al., 2009; Dong et al., 2015). In previous studies of TLE, ECN with both structural and functional impairments have been revealed in the patient group when compared with HCs. Such as, ventrolateral and dorsolateral prefrontal cortices and frontal operculum of ECN were demonstrated atrophy though voxel-based morphometry (VBM) analysis (Cataldi et al., 2013). Furthermore, PFC atrophy was recognized as a key factor in TLE with ECF deficits though VBM anaylsis either (Keller et al., 2009). Evidence from DTI research also supported this view, which found damaged white matter fiber connections between PFC and caudate had negative correlation with ECF performance (Kortte et al., 2002). FMRI study revealed FC decreased of ECN in TLE patients appearing with working memory impairment (Vlooswijk et al., 2011). In addition, PFC was also involved in TLE patients with cognitive impairment (Martin et al., 2000). These studies indicated that ECN played an important role in TLE with neuropsychological performance abnormalities. However, we did not find a significant difference in intra-network FC of the ECN and DMN among the three groups in our study. Unlike previous investigations, we divided patients into subgroups depending on ECF impairment, which allowed us to assess special FC changes associated with ECF impairment in TLE. Neuropsychological abnormalities of patients in our study were not only ECF dysfunction, but also included cognitive impairment as the result mentioned. It would be more conducive to remove other confounding factors potentially leading to alterations of RSNs, such as the impacts of the disease itself and other cognitive dysfuntion in exploring the ECF impairment specifically. Therefore, we made a comparison between patients with and without ECF impairment rather than that between patients and healthy volunteers. In addition, we found no significant difference of clinical features, such as age at seizure onset and seizure duration between G1 and G2 group. No significant intra-network FC difference was found between subgroups of patient that may due to the similar disruption patterns of each RSN in TLE with the similar clinical features. However, we only noted decreased inter-network FC between the ECN and DMN in patients with ECF impairment. We therefore inferred that ECF performance in TLE patients is also influenced by the functional integration of multiple networks (Miller and Cohen, 2001; Zamarian et al., 2011; Petersen and Sporns, 2015).

We employed the data-driven method of GIG-ICA to extract RSNs and calculate FC between different RSN pairs. Our findings were similar to a previous report of decreased connectivity between the DMN and ECN in left TLE patients (de Campos et al., 2016). The probable explanation was that left TLE patients are more susceptible to bilateral and diffuse functional organization changes due to both ictal and interictal activities (Voets et al., 2012; DeSalvo et al., 2014; Taylor et al., 2015), which may result in low interactions between large-scale networks and further lead to irreversible cognitive dysfunction (Hamberger et al., 2007; de Campos et al., 2016). Previous studies of FC between large-scale networks did not further subdivide the patients, which may have prevented them from noting differences. Our results show that FC between the ECN and DMN may help to identify TLE patients with ECF impairment. The DMN extracted from our data contained the bilateral MPFC and precuneus gyrus, and the MPFC is considered an especially important component (Broyd et al., 2009). The ECN from our data mainly covered the bilateral middle frontal gyrus and dorsolateral superior frontal gyrus, which were the major components of dorsolateral PFC (DLPFC) that was recognized as a core structure of the ECN (Seeley et al., 2007). PFC is a well-established structure for diverse information exchange and convergence to accomplish its role of cognitive control (Miller and Cohen, 2001). Thus, abnormal information transmission within the PFC could affect the function of whole PFC systems that may lead to ECF impairment. The MPFC was demonstrated closely related to emotion-based decision-making (Watanabe, 2016). The DLPFC was recognized as a central component to controlling behavior (Seeley et al., 2007). Since the MPFC and DLPFC are closely related to ECF (Royall et al., 2002), we assumed that decreased interaction between them may play a key role in ECF impairment due to TLE. In addition, previous studies indicated that working memory was a common basis of different executive functions among ECF components such as updating, shifting and inhibition (Miyake et al., 2000; Baddeley, 2001; Collette and Van der Linden, 2002). Besides, episodic memory capacity was proved positively correlated with DMN activity (McCormick et al., 2014). Decreased FC between the DMN and ECN represent reduced interaction between the two regions, so we concluded that damage to extra-temporal connections between two core RSNs involved in ECF and memory capacity may be another important element influencing ECF impairment in TLE (Keller and Roberts, 2008; Putcha et al., 2015).

Besides, we also found that FC between the ECN and DMN was significantly negatively correlated with RP, RE and RPE and positively correlated with CC in all TLE patients, suggesting that this FC pattern was positively correlated with ECF performance. Abnormal WCST scores are indicative of frontal lobe function damage, possibly impaired interactions between the MPFC of the DMN and the DLPFC of the ECN (Miller and Cohen, 2001), but this requires further study. However, our results demonstrated that FC between the ECN and DMN is significantly correlated with ECF performance. This FC pattern may be an important imaging feature reflecting alterations of RSNs in TLE patients with ECF impairment.

Finally, FC between the DMN and ECN was not significantly correlated with MoCA or IQ test scores in either TLE group in our study, suggesting that the abnormal FC was due to the disease itself, rather than other common factors of the patients.

### Limitations

Several limitations should be noted in our study. First, our design did not allow us to control for confounding effects of antiepileptic drugs, which may affect ECF performance. Second, we only included patient with left-hemisphere TLE, it would be more comprehensive research to include subjects with right-hemisphere TLE. Third, our study is based on group-level analysis, making it difficult to apply our findings to individual patients. The fourth, we did not use Bonferroni correction to control for multiple comparisons on evaluating FC with WSCT correlations. In the future, analyzing TLE patients with ECF on an individual level will be more helpful to guide clinical management.

## Conclusion

Our results show that ECF performance in patients with unilateral TLE is associated with functional integration involving multiple brain regions beyond the diseased temporal lobe. Decreased FC between the DMN and ECN was uncovered in TLE with ECF impairment, suggesting that this alteration may be a specific feature of ECF impairment due to epileptic events. Disturbed interaction between the DMN and ECN may be an underlying pathological mechanism of ECF impairment in patients with intractable unilateral left TLE.

## Author Contributions

CZ and HY: conception/design of the work; acquisition, analysis and interpretation of data for the work; drafting of the work; final approval of the version to be published and agreement to be accountable for all aspects of the work. WQ, ZQ and CL: data analysis, data for the work; drafting of the work; final approval of the version to be published and agreement to be accountable for all aspects of the work. NC and KL: design of the work; revision of the work; final approval of the version to be published and agreement to be accountable for all aspects of the work.

## Conflict of Interest Statement

The authors declare that the research was conducted in the absence of any commercial or financial relationships that could be construed as a potential conflict of interest.

## Footnotes

^1^https://www.nitrc.org/projects/gift

## Abbreviations

BOLD: blood oxygen level-dependent
CC: categories completed
DTI: diffusion tensor imaging
ECF: executive control function
ECN: executive control network
FC: functional connectivity
FD: framewise displacement
FDR: false discovery rate
FLAIR: fluid-attenuated inverse recovery
FPN: frontoparietal network
GIG-ICA: group-information-guided independent component analysis
GLM: general linear model
HC: healthy control
HS: hippocampal sclerosis
ICs: independent components
IQ: intelligence quotient
MoCA: Montreal Cognitive Assessment
NRPE: nonperseverative errors
PFC: prefrontal cortex
RE: response errors
RP: perseverative responses
RPE: perseverative errors
rs-fMRI: resting-state fMRI
RSN: resting-state network
SMN: sensorimotor-related network
SPM: statistical parametric mapping
TLE: temporal lobe epilepsy
VBM: voxel-based morphometry
VN: visual network
VSN: visuospatial network
WAIS: Wechsler Adult Intelligence Scale
WCST: Wisconsin Card Sorting Test.

## References

[B1] Baddeley A. D. (2001). Is working memory still working? Am. Psychol. 56, 851–864. 10.1037/0003-066x.56.11.851 11785152

[B2] Baggio H. C. Segura B. Junque C. (2015a). Resting-state functional brain networks in Parkinson’s disease. CNS Neurosci. Ther. 21, 793–801. 10.1111/cns.12417 26224057 PMC6093256

[B3] Baggio H. C. Segura B. Sala-Llonch R. Marti M. J. Valldeoriola F. Compta Y.. (2015b). Cognitive impairment and resting-state network connectivity in Parkinson’s disease. Hum. Brain Mapp. 36, 199–212. 10.1002/hbm.22622 25164875 PMC6869118

[B4] Bell B. Lin J. J. Seidenberg M. Hermann B. (2011). The neurobiology of cognitive disorders in temporal lobe epilepsy. Nat. Rev. Neurol. 7, 154–164. 10.1038/nrneurol.2011.3 21304484 PMC3856217

[B5] Bernhardt B. C. Chen Z. He Y. Evans A. C. Bernasconi N. (2011). Graph-theoretical analysis reveals disrupted small-world organization of cortical thickness correlation networks in temporal lobe epilepsy. Cereb. Cortex 21, 2147–2157. 10.1093/cercor/bhq291 21330467

[B6] Biswal B. Yetkin F. Z. Haughton V. M. Hyde J. S. (1995). Functional connectivity in the motor cortex of resting human brain using echo-planar MRI. Magn. Reson. Med. 34, 537–541. 10.1002/mrm.1910340409 8524021

[B7] Bossong M. G. Jansma J. M. van Hell H. H. Jager G. Kahn R. S. Ramsey N. F. (2013). Default mode network in the effects of ∆9-Tetrahydrocannabinol (THC) on human executive function. PLoS One 8:e70074. 10.1371/journal.pone.0070074 23936144 PMC3729458

[B8] Brewer J. A. Worhunsky P. D. Gray J. R. Tang Y. Y. Weber J. Kober H. (2011). Meditation experience is associated with differences in default mode network activity and connectivity. Proc. Natl. Acad. Sci. U S A 108, 20254–20259. 10.1073/pnas.1112029108 22114193 PMC3250176

[B9] Browndyke J. N. Berger M. Harshbarger T. B. Smith P. J. White W. Bisanar T. L.. (2017). Resting-state functional connectivity and cognition after major cardiac surgery in older adults without preoperative cognitive impairment: preliminary findings. J. Am. Geriatr. Soc. 65, e6–e12. 10.1111/jgs.14534 27858963 PMC5258858

[B10] Broyd S. J. Demanuele C. Debener S. Helps S. K. James C. J. Sonuga-Barke E. J. (2009). Default-mode brain dysfunction in mental disorders: a systematic review. Neurosci. Biobehav. Rev. 33, 279–296. 10.1016/j.neubiorev.2008.09.002 18824195

[B11] Cataldi M. Avoli M. de Villers-Sidani E. (2013). Resting state networks in temporal lobe epilepsy. Epilepsia 54, 2048–2059. 10.1111/epi.12400 24117098 PMC4880458

[B12] Chai X. J. Castañón A. N. Ongür D. Whitfield-Gabrieli S. (2012). Anticorrelations in resting state networks without global signal regression. Neuroimage 59, 1420–1428. 10.1016/j.neuroimage.2011.08.048 21889994 PMC3230748

[B13] Collette F. Van der Linden M. (2002). Brain imaging of the central executive component of working memory. Neurosci. Biobehav. Rev. 26, 105–125. 10.1016/s0149-7634(01)00063-x 11856556

[B14] Corcoran R. Upton D. (1993). A role for the hippocampus in card sorting? Cortex 29, 293–304. 10.1016/s0010-9452(13)80182-7 8348826

[B15] Crittenden B. M. Mitchell D. J. Duncan J. (2015). Recruitment of the default mode network during a demanding act of executive control. Elife 4:e06481. 10.7554/eLife.06481 25866927 PMC4427863

[B16] de Campos B. M. Coan A. C. Lin Yasuda C. Casseb R. F. Cendes F. (2016). Large-scale brain networks are distinctly affected in right and left mesial temporal lobe epilepsy. Hum. Brain Mapp. 37, 3137–3152. 10.1002/hbm.23231 27133613 PMC5074272

[B17] DeSalvo M. N. Douw L. Tanaka N. Reinsberger C. Stufflebeam S. M. (2014). Altered structural connectome in temporal lobe epilepsy. Radiology 270, 842–848. 10.1148/radiol.13131044 24475828 PMC4263659

[B18] Dong G. Lin X. Potenza M. N. (2015). Decreased functional connectivity in an executive control network is related to impaired executive function in Internet gaming disorder. Prog. Neuropsychopharmacol. Biol. Psychiatry 57, 76–85. 10.1016/j.pnpbp.2014.10.012 25445475 PMC4473260

[B19] Du Y. Fan Y. (2013). Group information guided ICA for fMRI data analysis. Neuroimage 69, 157–197. 10.1016/j.neuroimage.2012.11.008 23194820

[B20] Du Y. Pearlson G. D. Liu J. Sui J. Yu Q. He H.. (2015). A group ICA based framework for evaluating resting fMRI markers when disease categories are unclear: application to schizophrenia, bipolar, and schizoaffective disorders. Neuroimage 122, 272–280. 10.1016/j.neuroimage.2015.07.054 26216278 PMC4618037

[B21] Fazel S. Wolf A. Långström N. Newton C. R. Lichtenstein P. (2013). Premature mortality in epilepsy and the role of psychiatric comorbidity: a total population study. Lancet 382, 1646–1654. 10.1016/S0140-6736(13)60899-5 23883699 PMC3899026

[B22] Giovagnoli A. R. (2001). Relation of sorting impairment to hippocampal damage in temporal lobe epilepsy. Neuropsychologia 39, 140–150. 10.1016/s0028-3932(00)00104-4 11163372

[B23] Grafman J. Litvan I. (1999). Importance of deficits in executive functions. Lancet 354, 1921–1923. 10.1016/s0140-6736(99)90438-5 10622291

[B24] Hamberger M. J. Palmese C. A. Scarmeas N. Weintraub D. Choi H. Hirsch L. J. (2007). A randomized, double-blind, placebo-controlled trial of donepezil to improve memory in epilepsy. Epilepsia 48, 1283–1291. 10.1111/j.1528-1167.2007.01114.x 17484756

[B25] Ibrahim G. M. Morgan B. R. Lee W. Smith M. L. Donner E. J. Wang F.. (2014). Impaired development of intrinsic connectivity networks in children with medically intractable localization-related epilepsy. Hum. Brain Mapp. 35, 5686–5700. 10.1002/hbm.22580 24976288 PMC6869397

[B26] Jiang Y. Xia J. Li S. Chen J. Wang P. Chen Q. (2017). Neural dynamics underlying varying attentional control facing invariant cognitive task upon invariant stimuli. Neuroscience 353, 133–146. 10.1016/j.neuroscience.2017.04.023 28450264

[B27] Keezer M. R. Sisodiya S. M. Sander J. W. (2016). Comorbidities of epilepsy: current concepts and future perspectives. Lancet Neurol. 15, 106–115. 10.1016/S1474-4422(15)00225-2 26549780

[B29] Keller S. S. Baker G. Downes J. J. Roberts N. (2009). Quantitative MRI of the prefrontal cortex and executive function in patients with temporal lobe epilepsy. Epilepsy Behav. 15, 186–195. 10.1016/j.yebeh.2009.03.005 19286475

[B28] Keller S. S. Roberts N. (2008). Voxel-based morphometry of temporal lobe epilepsy: an introduction and review of the literature. Epilepsia 49, 741–757. 10.1111/j.1528-1167.2007.01485.x 18177358

[B30] Kim C. H. Lee S. A. Yoo H. J. Kang J. K. Lee J. K. (2007). Executive performance on the Wisconsin Card Sorting Test in mesial temporal lobe epilepsy. Eur. Neurol. 57, 39–46. 10.1159/000097009 17108694

[B31] Kortte K. B. Horner M. D. Windham W. K. (2002). The trail making test, part B: cognitive flexibility or ability to maintain set? Appl. Neuropsychol. 9, 106–109. 10.1207/s15324826an0902_5 12214820

[B32] Kucukboyaci N. E. Girard H. M. Hagler D. J. Jr. Kuperman J. Tecoma E. S. Iragui V. J.. (2012). Role of frontotemporal fiber tract integrity in task-switching performance of healthy controls and patients with temporal lobe epilepsy. J. Int. Neuropsychol. Soc. 18, 57–67. 10.1017/S1355617711001391 22014246 PMC3482626

[B33] Leyden K. M. Kucukboyaci N. E. Puckett O. K. Lee D. Loi R. Q. Paul B.. (2015). What does diffusion tensor imaging (DTI) tell us about cognitive networks in temporal lobe epilepsy? Quant. Imaging Med. Surg. 5, 247–263. 10.3978/j.issn.2223-4292.2015.02.01 25853083 PMC4379319

[B34] Liang X. Zou Q. He Y. Yang Y. (2013). Coupling of functional connectivity and regional cerebral blood flow reveals a physiological basis for network hubs of the human brain. Proc. Natl. Acad. Sci. U S A 110, 1929–1934. 10.1073/pnas.1214900110 23319644 PMC3562840

[B35] Lin J. J. Mula M. Hermann B. P. (2012). Uncovering the neurobehavioural comorbidities of epilepsy over the lifespan. Lancet 380, 1180–1192. 10.1016/s0140-6736(12)61455-x 23021287 PMC3838617

[B36] Liu F. Guo W. Fouche J. P. Wang Y. Wang W. Ding J.. (2015). Multivariate classification of social anxiety disorder using whole brain functional connectivity. Brain Struct. Funct. 220, 101–115. 10.1007/s00429-013-0641-4 24072164

[B37] Ma Y. Shaik M. A. Kozberg M. G. Kim S. H. Portes J. P. Timerman D.. (2016). Resting-state hemodynamics are spatiotemporally coupled to synchronized and symmetric neural activity in excitatory neurons. Proc. Natl. Acad. Sci. U S A 113, E8463–e8471. 10.1073/pnas.1525369113 27974609 PMC5206542

[B39] Mantini D. Perrucci M. G. Del Gratta C. Romani G. L. Corbetta M. (2007). Electrophysiological signatures of resting state networks in the human brain. Proc. Natl. Acad. Sci. U S A 104, 13170–13175. 10.1073/pnas.0700668104 17670949 PMC1941820

[B40] Martin R. C. Sawrie S. M. Gilliam F. G. Palmer C. A. Faught E. Morawetz R. B.. (2000). Wisconsin Card Sorting performance in patients with temporal lobe epilepsy: clinical and neuroanatomical correlates. Epilepsia 41, 1626–1632. 10.1111/j.1528-1167.2000.01626.x 11114222

[B41] McCormick C. Protzner A. B. Barnett A. J. Cohn M. Valiante T. A. McAndrews M. P. (2014). Linking DMN connectivity to episodic memory capacity: what can we learn from patients with medial temporal lobe damage? Neuroimage Clin. 5, 188–196. 10.1016/j.nicl.2014.05.008 25068108 PMC4110351

[B42] Miller E. K. Cohen J. D. (2001). An integrative theory of prefrontal cortex function. Annu. Rev. Neurosci. 24, 167–202. 10.1146/annurev.neuro.24.1.167 11283309

[B43] Miyake A. Friedman N. P. Emerson M. J. Witzki A. H. Howerter A. Wager T. D. (2000). The unity and diversity of executive functions and their contributions to complex “Frontal Lobe” tasks: a latent variable analysis. Cogn. Psychol. 41, 49–100. 10.1006/cogp.1999.0734 10945922

[B44] Mohan A. Roberto A. J. Mohan A. Lorenzo A. Jones K. Carney M. J.. (2016). The significance of the default mode network (DMN) in neurological and neuropsychiatric disorders: a review. Yale J. Biol. Med. 89, 49–57. 27505016 PMC4797836

[B45] Oser N. Hubacher M. Specht K. Datta A. N. Weber P. Penner I. K. (2014). Default mode network alterations during language task performance in children with benign epilepsy with centrotemporal spikes (BECTS). Epilepsy Behav. 33, 12–17. 10.1016/j.yebeh.2014.01.008 24583653

[B46] Pereira L. Airan R. D. Fishman A. Pillai J. J. Kansal K. Onyike C. U.. (2017). Resting-state functional connectivity and cognitive dysfunction correlations in spinocerebelellar ataxia type 6 (SCA6). Hum. Brain Mapp. 38, 3001–3010. 10.1002/hbm.23568 28295805 PMC6866919

[B47] Petersen S. E. Sporns O. (2015). Brain networks and cognitive architectures. Neuron 88, 207–219. 10.1016/j.neuron.2015.09.027 26447582 PMC4598639

[B48] Power J. D. Schlaggar B. L. Petersen S. E. (2014). Studying brain organization via spontaneous fMRI signal. Neuron 84, 681–696. 10.1016/j.neuron.2014.09.007 25459408 PMC4254503

[B49] Putcha D. Ross R. S. Cronin-Golomb A. Janes A. C. Stern C. E. (2015). Altered intrinsic functional coupling between core neurocognitive networks in Parkinson’s disease. Neuroimage Clin. 7, 449–455. 10.1016/j.nicl.2015.01.012 25685711 PMC4320252

[B50] Raichle M. E. (2015). The brain’s default mode network. Annu. Rev. Neurosci. 38, 433–447. 10.1146/annurev-neuro-071013-014030 25938726

[B51] Royall D. R. Lauterbach E. C. Cummings J. L. Reeve A. Rummans T. A. Kaufer D. I.. (2002). Executive control function: a review of its promise and challenges for clinical research. A report from the Committee on Research of the American Neuropsychiatric Association. J. Neuropsychiatry Clin. Neurosci. 14, 377–405. 10.1176/appi.neuropsych.14.4.377 12426407

[B52] Saad Z. S. Gotts S. J. Murphy K. Chen G. Jo H. J. Martin A.. (2012). Trouble at rest: how correlation patterns and group differences become distorted after global signal regression. Brain Connect. 2, 25–32. 10.1089/brain.2012.0080 22432927 PMC3484684

[B53] Scholvinck M. L. Maier A. Ye F. Q. Duyn J. H. Leopold D. A. (2010). Neural basis of global resting-state fMRI activity. Proc. Natl. Acad. Sci. U S A 107, 10238–10243. 10.1073/pnas.0913110107 20439733 PMC2890438

[B54] Seeley W. W. Menon V. Schatzberg A. F. Keller J. Glover G. H. Kenna H.. (2007). Dissociable intrinsic connectivity networks for salience processing and executive control. J. Neurosci. 27, 2349–2356. 10.1523/JNEUROSCI.5587-06.2007 17329432 PMC2680293

[B55] Sherman L. E. Rudie J. D. Pfeifer J. H. Masten C. L. McNealy K. Dapretto M. (2014). Development of the default mode and central executive networks across early adolescence: a longitudinal study. Dev. Cogn. Neurosci. 10, 148–159. 10.1016/j.dcn.2014.08.002 25282602 PMC4854607

[B56] Smith S. M. Fox P. T. Miller K. L. Glahn D. C. Fox P. M. Mackay C. E.. (2009). Correspondence of the brain’s functional architecture during activation and rest. Proc. Natl. Acad. Sci. U S A 106, 13040–13045. 10.1073/pnas.0905267106 19620724 PMC2722273

[B57] Song J. Qin W. Liu Y. Duan Y. Liu J. He X.. (2013). Aberrant functional organization within and between resting-state networks in AD. PLoS One 8:e63727. 10.1371/journal.pone.0063727 23667665 PMC3647055

[B58] Stam C. J. de Haan W. Daffertshofer A. Jones B. F. Manshanden I. van Cappellen van Walsum A. M.. (2009). Graph theoretical analysis of magnetoencephalographic functional connectivity in Alzheimer’s disease. Brain 132, 213–224. 10.1093/brain/awn262 18952674

[B59] Takaya S. Hanakawa T. Hashikawa K. Ikeda A. Sawamoto N. Nagamine T.. (2006). Prefrontal hypofunction in patients with intractable mesial temporal lobe epilepsy. Neurology 67, 1674–1676. 10.1212/01.wnl.0000242628.26978.e2 17101904

[B60] Taylor P. N. Han C. E. Schoene-Bake J. C. Weber B. Kaiser M. (2015). Structural connectivity changes in temporal lobe epilepsy: spatial features contribute more than topological measures. Neuroimage Clin. 8, 322–328. 10.1016/j.nicl.2015.02.004 26106557 PMC4473265

[B61] Tuchscherer V. Seidenberg M. Pulsipher D. Lancaster M. Guidotti L. Hermann B. (2010). Extrahippocampal integrity in temporal lobe epilepsy and cognition: thalamus and executive functioning. Epilepsy Behav. 17, 478–482. 10.1016/j.yebeh.2010.01.019 20185373

[B62] Vaughan D. N. Rayner G. Tailby C. Jackson G. D. (2016). MRI-negative temporal lobe epilepsy: a network disorder of neocortical connectivity. Neurology 87, 1934–1942. 10.1212/WNL.0000000000003289 27694267

[B63] Vlooswijk M. C. Jansen J. F. Jeukens C. R. Majoie H. J. Hofman P. A. de Krom M. C.. (2011). Memory processes and prefrontal network dysfunction in cryptogenic epilepsy. Epilepsia 52, 1467–1475. 10.1111/j.1528-1167.2011.03108.x 21635235

[B64] Voets N. L. Beckmann C. F. Cole D. M. Hong S. Bernasconi A. Bernasconi N. (2012). Structural substrates for resting network disruption in temporal lobe epilepsy. Brain 135, 2350–2357. 10.1093/brain/aws137 22669081

[B65] Vollmar C. O’Muircheartaigh J. Barker G. J. Symms M. R. Thompson P. Kumari V.. (2011). Motor system hyperconnectivity in juvenile myoclonic epilepsy: a cognitive functional magnetic resonance imaging study. Brain 134, 1710–1719. 10.1093/brain/awr098 21616969 PMC3102244

[B67] Wang X. Q. Iang S. Y. Lu H. Ma L. Mao Y. L. Yang F. (2007). Executive function impairment in patients with temporal lobe epilepsy: neuropsychological and diffusion-tensor imaging study. Zhonghua Yi Xue Za Zhi 87, 3183–3187. 18399110

[B66] Wang C. Qin W. Zhang J. Tian T. Li Y. Meng L.. (2014). Altered functional organization within and between resting-state networks in chronic subcortical infarction. J. Cereb. Blood Flow Metab. 34, 597–605. 10.1038/jcbfm.2013.238 24398939 PMC3982082

[B68] Watanabe M. (2016). Emotional and motivational functions of the prefrontal cortex. Brain Nerve 68, 1291–1299. 10.11477/mf.1416200593 27852020

[B69] Widjaja E. Skocic J. Go C. Snead O. C. Mabbott D. Smith M. L. (2013). Abnormal white matter correlates with neuropsychological impairment in children with localization-related epilepsy. Epilepsia 54, 1065–1073. 10.1111/epi.12208 23650911 PMC3867411

[B70] Wu J. Quinlan E. B. Dodakian L. McKenzie A. Kathuria N. Zhou R. J.. (2015). Connectivity measures are robust biomarkers of cortical function and plasticity after stroke. Brain 138, 2359–2369. 10.1093/brain/awv156 26070983 PMC4840951

[B71] Yan C. G. Cheung B. Kelly C. Colcombe S. Craddock R. C. Di Martino A.. (2013). A comprehensive assessment of regional variation in the impact of head micromovements on functional connectomics. Neuroimage 76, 183–201. 10.1016/j.neuroimage.2013.03.004 23499792 PMC3896129

[B72] Yan C. G. Wang X. D. Zuo X. N. Zang Y. F. (2016). DPABI: data processing and analysis for (resting-state) brain imaging. Neuroinformatics 14, 339–351. 10.1007/s12021-016-9299-4 27075850

[B73] Yang G. J. Murray J. D. Repovs G. Cole M. W. Savic A. Glasser M. F.. (2014). Altered global brain signal in schizophrenia. Proc. Natl. Acad. Sci. U S A 111, 7438–7443. 10.1073/pnas.1405289111 24799682 PMC4034208

[B74] Yeo B. T. Krienen F. M. Sepulcre J. Sabuncu M. R. Lashkari D. Hollinshead M.. (2011). The organization of the human cerebral cortex estimated by intrinsic functional connectivity. J. Neurophysiol. 106, 1125–1165. 10.1152/jn.00338.2011 21653723 PMC3174820

[B75] Zamarian L. Trinka E. Bonatti E. Kuchukhidze G. Bodner T. Benke T.. (2011). Executive functions in chronic mesial temporal lobe epilepsy. Epilepsy Res. Treat. 2011:596174. 10.1155/2011/596174 22937233 PMC3428608

[B76] Zhang Z. Lu G. Zhong Y. Tan Q. Liao W. Chen Z.. (2009). Impaired perceptual networks in temporal lobe epilepsy revealed by resting fMRI. J. Neurol. 256, 1705–1713. 10.1007/s00415-009-5187-2 19488674

