# Supplementary material for: Characteristics of Resting-State Functional Connectivity in Intractable Unilateral Temporal Lobe Epilepsy Patients with Impaired Executive Control Function
Source: Front Hum Neurosci. 2017 Dec 13;11:609. doi: 10.3389/fnhum.2017.00609 (PMC5770650; doi:10.3389/fnhum.2017.00609)
Supplement: Supplementary file 1 [file Data_Sheet_1.doc]

T-TEST GROUPS=GROUP(2 3)
  /MISSING=ANALYSIS
  /VARIABLES=gender
  /CRITERIA=CI(.9500).

T-Test


[DataSet1] C:\Users\zhangchao\Desktop\frontiers\FC_P.sav

Group Statistics	
	GROUP	N	Mean	Std. Deviation	Std. Error Mean	
gender	2	18	1.6667	.48507	.11433	
	3	22	1.5455	.50965	.10866	


Independent Samples Test	
		Levene's Test for Equality of Variances	t-test for Equality of Means	
		F	Sig.	t	df	Sig. (2-tailed)	Mean Difference	Std. Error Difference	95% Confidence Interval of the Difference	
									Lower	Upper	
gender	Equal variances assumed	2.032	.162	.765	38	.449	.12121	.15853	-.19971	.44214	
	Equal variances not assumed			.768	37.086	.447	.12121	.15773	-.19835	.44078	
