# Supplementary material for: Characteristics of Resting-State Functional Connectivity in Intractable Unilateral Temporal Lobe Epilepsy Patients with Impaired Executive Control Function
Source: Front Hum Neurosci. 2017 Dec 13;11:609. doi: 10.3389/fnhum.2017.00609 (PMC5770650; doi:10.3389/fnhum.2017.00609)
Supplement: Supplementary file 3 [file Data_Sheet_3.doc]

T-Test

[DataSet0] C:\Users\zhangchao\Desktop\frontiers\FC_P.sav

Group Statistics	
	GROUP	N	Mean	Std. Deviation	Std. Error Mean	
FC	2	18	-.1233	.25011	.05895	
	3	22	-.4723	.19618	.04182	


Independent Samples Test	
		Levene's Test for Equality of Variances	t-test for Equality of Means	
		F	Sig.	t	df	Sig. (2-tailed)	Mean Difference	Std. Error Difference	95% Confidence Interval of the Difference	
									Lower	Upper	
FC	Equal variances assumed	1.279	.265	4.947	38	.000	.34894	.07053	.20615	.49173	
	Equal variances not assumed			4.828	31.883	.000	.34894	.07228	.20169	.49619	
