# Supplementary material for: Characteristics of Resting-State Functional Connectivity in Intractable Unilateral Temporal Lobe Epilepsy Patients with Impaired Executive Control Function
Source: Front Hum Neurosci. 2017 Dec 13;11:609. doi: 10.3389/fnhum.2017.00609 (PMC5770650; doi:10.3389/fnhum.2017.00609)
Supplement: Supplementary file 6 [file Data_Sheet_6.doc]

UNIANOVA FC BY GROUP WITH gender age edu IQ meanFD
  /METHOD=SSTYPE(3)
  /INTERCEPT=INCLUDE
  /EMMEANS=TABLES(GROUP) WITH(gender=MEAN age=MEAN edu=MEAN IQ=MEAN meanFD=MEAN) COMPARE ADJ(BONFERRONI)
  /CRITERIA=ALPHA(.05)
  /DESIGN=gender age edu IQ meanFD GROUP.

Univariate Analysis of Variance

Notes	
Output Created	01-Ê®ÔÂ-2017 02Ê±00·Ö34Ãë	
Comments		
Input	Data	C:\Users\zhangchao\Desktop\frontiers\FC_P.sav	
	Active Dataset	DataSet1	
	Filter	<none>	
	Weight	<none>	
	Split File	<none>	
	N of Rows in Working Data File	63	
Missing Value Handling	Definition of Missing	User-defined missing values are treated as missing.	
	Cases Used	Statistics are based on all cases with valid data for all variables in the model.	
Syntax	UNIANOVA FC BY GROUP WITH gender age edu IQ meanFD
  /METHOD=SSTYPE(3)
  /INTERCEPT=INCLUDE
  /EMMEANS=TABLES(GROUP) WITH(gender=MEAN age=MEAN edu=MEAN IQ=MEAN meanFD=MEAN) COMPARE ADJ(BONFERRONI)
  /CRITERIA=ALPHA(.05)
  /DESIGN=gender age edu IQ meanFD GROUP.
	
Resources	Processor Time	00Ê±00·Ö00Ãë	
	Elapsed Time	00Ê±00·Ö00Ãë	


[DataSet1] C:\Users\zhangchao\Desktop\frontiers\FC_P.sav

Between-Subjects Factors	
		Value Label	N	
GROUP	1	HC	23	
	2	G1	18	
	3	G2	22	


Tests of Between-Subjects Effects	
Dependent Variable:FC					
Source	Type III Sum of Squares	df	Mean Square	F	Sig.	
Corrected Model	2.012a	7	.287	5.869	.000	
Intercept	.016	1	.016	.325	.571	
gender	.001	1	.001	.026	.873	
age	.002	1	.002	.042	.838	
edu	.003	1	.003	.061	.806	
IQ	.048	1	.048	.984	.326	
meanFD	.072	1	.072	1.462	.232	
GROUP	1.579	2	.790	16.125	.000	
Error	2.693	55	.049			
Total	8.272	63				
Corrected Total	4.705	62				
a. R Squared = .428 (Adjusted R Squared = .355)			


Estimated Marginal Means

GROUP

Estimates	
Dependent Variable:FC			
GROUP	Mean	Std. Error	95% Confidence Interval	
			Lower Bound	Upper Bound	
HC	-.100a	.050	-.200	.000	
G1	-.116a	.053	-.223	-.009	
G2	-.482a	.051	-.584	-.379	
a. Covariates appearing in the model are evaluated at the following values: gender = 1.5556, age = 26.0159, edu = 12.3492, IQ = 92.0000, meanFD = .1202.	


Pairwise Comparisons	
Dependent Variable:FC					
(I) GROUP	(J) GROUP	Mean Difference (I-J)	Std. Error	Sig.a	95% Confidence Interval for Differencea	
					Lower Bound	Upper Bound	
HC	G1	.016	.074	1.000	-.166	.197	
	G2	.381*	.076	.000	.193	.570	
G1	HC	-.016	.074	1.000	-.197	.166	
	G2	.366*	.075	.000	.182	.550	
G2	HC	-.381*	.076	.000	-.570	-.193	
	G1	-.366*	.075	.000	-.550	-.182	
Based on estimated marginal means				
a. Adjustment for multiple comparisons: Bonferroni.		
*. The mean difference is significant at the .05 level.			


Univariate Tests	
Dependent Variable:FC					
	Sum of Squares	df	Mean Square	F	Sig.	
Contrast	1.579	2	.790	16.125	.000	
Error	2.693	55	.049			
The F tests the effect of GROUP. This test is based on the linearly independent pairwise comparisons among the estimated marginal means.	
