# Supplementary material for: Characteristics of Resting-State Functional Connectivity in Intractable Unilateral Temporal Lobe Epilepsy Patients with Impaired Executive Control Function
Source: Front Hum Neurosci. 2017 Dec 13;11:609. doi: 10.3389/fnhum.2017.00609 (PMC5770650; doi:10.3389/fnhum.2017.00609)
Supplement: Supplementary file 7 [file Data_Sheet_7.doc]

GET
  FILE='C:\Users\zhangchao\Desktop\frontiers\adult_spss.sav'.
DATASET NAME DataSet0 WINDOW=FRONT.
UNIANOVA FC BY G WITH gender age edu IQ meanFD
  /METHOD=SSTYPE(3)
  /INTERCEPT=INCLUDE
  /EMMEANS=TABLES(G) WITH(gender=MEAN age=MEAN edu=MEAN IQ=MEAN meanFD=MEAN) COMPARE ADJ(BONFERRONI)
  /CRITERIA=ALPHA(.05)
  /DESIGN=gender age edu IQ meanFD G.

Univariate Analysis of Variance

Notes	
Output Created	01-十月-2017 02时08分37秒	
Comments		
Input	Data	C:\Users\zhangchao\Desktop\frontiers\adult_spss.sav	
	Active Dataset	DataSet1	
	Filter	<none>	
	Weight	<none>	
	Split File	<none>	
	N of Rows in Working Data File	59	
Missing Value Handling	Definition of Missing	User-defined missing values are treated as missing.	
	Cases Used	Statistics are based on all cases with valid data for all variables in the model.	
Syntax	UNIANOVA FC BY G WITH gender age edu IQ meanFD
  /METHOD=SSTYPE(3)
  /INTERCEPT=INCLUDE
  /EMMEANS=TABLES(G) WITH(gender=MEAN age=MEAN edu=MEAN IQ=MEAN meanFD=MEAN) COMPARE ADJ(BONFERRONI)
  /CRITERIA=ALPHA(.05)
  /DESIGN=gender age edu IQ meanFD G.
	
Resources	Processor Time	00时00分00秒	
	Elapsed Time	00时00分00秒	


[DataSet1] C:\Users\zhangchao\Desktop\frontiers\adult_spss.sav

Between-Subjects Factors	
		Value Label	N	
G	1	HC	23	
	2	G1	14	
	3	G2	20	


Tests of Between-Subjects Effects	
Dependent Variable:FC					
Source	Type III Sum of Squares	df	Mean Square	F	Sig.	
Corrected Model	1.999a	7	.286	5.684	.000	
Intercept	.047	1	.047	.931	.339	
gender	.000	1	.000	.004	.953	
age	.005	1	.005	.102	.751	
edu	.001	1	.001	.024	.878	
IQ	.089	1	.089	1.767	.190	
meanFD	.056	1	.056	1.120	.295	
G	1.689	2	.844	16.805	.000	
Error	2.462	49	.050			
Total	7.749	57				
Corrected Total	4.461	56				
a. R Squared = .448 (Adjusted R Squared = .369)			


Estimated Marginal Means

G

Estimates	
Dependent Variable:FC			
G	Mean	Std. Error	95% Confidence Interval	
			Lower Bound	Upper Bound	
HC	-.100a	.050	-.201	.001	
G1	-.098a	.061	-.221	.026	
G2	-.501a	.054	-.610	-.393	
a. Covariates appearing in the model are evaluated at the following values: gender = 1.5614, age = 27.0877, edu = 12.5263, IQ = 92.3509, meanFD = .1228.	


Pairwise Comparisons	
Dependent Variable:FC					
(I) G	(J) G	Mean Difference (I-J)	Std. Error	Sig.a	95% Confidence Interval for Differencea	
					Lower Bound	Upper Bound	
HC	G1	-.002	.080	1.000	-.202	.197	
	G2	.401*	.079	.000	.207	.596	
G1	HC	.002	.080	1.000	-.197	.202	
	G2	.404*	.082	.000	.201	.607	
G2	HC	-.401*	.079	.000	-.596	-.207	
	G1	-.404*	.082	.000	-.607	-.201	
Based on estimated marginal means				
a. Adjustment for multiple comparisons: Bonferroni.		
*. The mean difference is significant at the .05 level.			


Univariate Tests	
Dependent Variable:FC					
	Sum of Squares	df	Mean Square	F	Sig.	
Contrast	1.689	2	.844	16.805	.000	
Error	2.462	49	.050			
The F tests the effect of G. This test is based on the linearly independent pairwise comparisons among the estimated marginal means.	
