# Supplementary material for: Characteristics of Resting-State Functional Connectivity in Intractable Unilateral Temporal Lobe Epilepsy Patients with Impaired Executive Control Function
Source: Front Hum Neurosci. 2017 Dec 13;11:609. doi: 10.3389/fnhum.2017.00609 (PMC5770650; doi:10.3389/fnhum.2017.00609)
Supplement: Supplementary file 8 [file Image_1.pdf]

Supplementary Figures

Impaired Executive Control Function in Intractable Unilateral Temporal Lobe Epilepsy: Revealed by Extrinsic Brain Network Connectivity

Chao Zhang,[1,2+] **Hongyu Yang,**[1,2+] **Wen Qin,**[3] **Chang Liu,**[4] **Zhigang Qi,**[1,2]

***Correspondence**: Nan Chen

E-mail address: chenzen8057@sina.com

***Correspondence may also be**: Kuncheng Li

E-mail address: cjr.likuncheng@vip.163.com

+ contributed equally to this work

1 Supplementary Figures

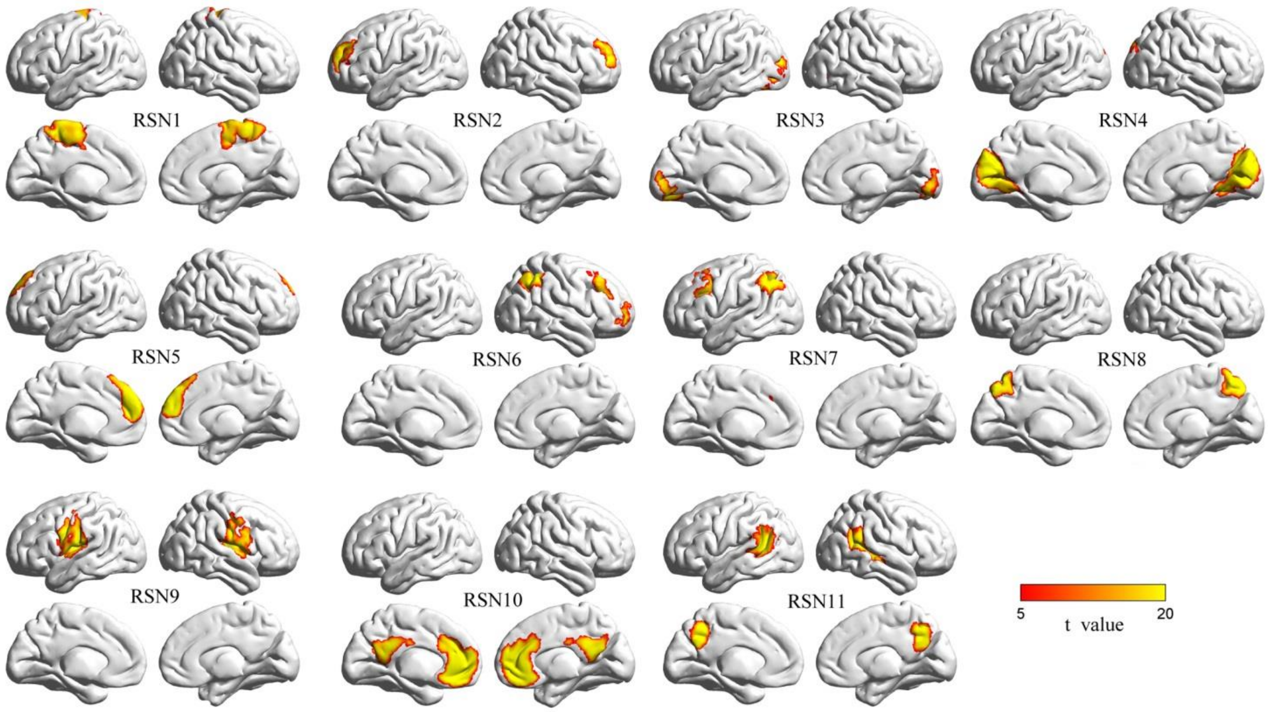

Supplementary Figure 1. Three-dimensional images made by BrainNet Viewer. Eleven RSNs identified by GIG-ICA across all subjects. Functional maps were showed on the lateral and medial surfaces of bilateral hemisphere. The color scale represents the t values in each RSN. RSN1, dorsal

sensorimotor network (dSMN); RSN2, executive control network (ECN); RSN3, occipital pole visual network (pVN); RSN4, medial visual network (mVN); RSN5, anterior default-mode related network (aDMN); RSN6, left frontoparietal network (lFPN); RSN7, right frontoparietal network (rFPN); RSN8, visuospatial network (VSN); RSN9, ventral sensorimotor related network (vSMN); RSN10, default-mode network (DMN); RSN11, posterior default-mode network (pDMN).

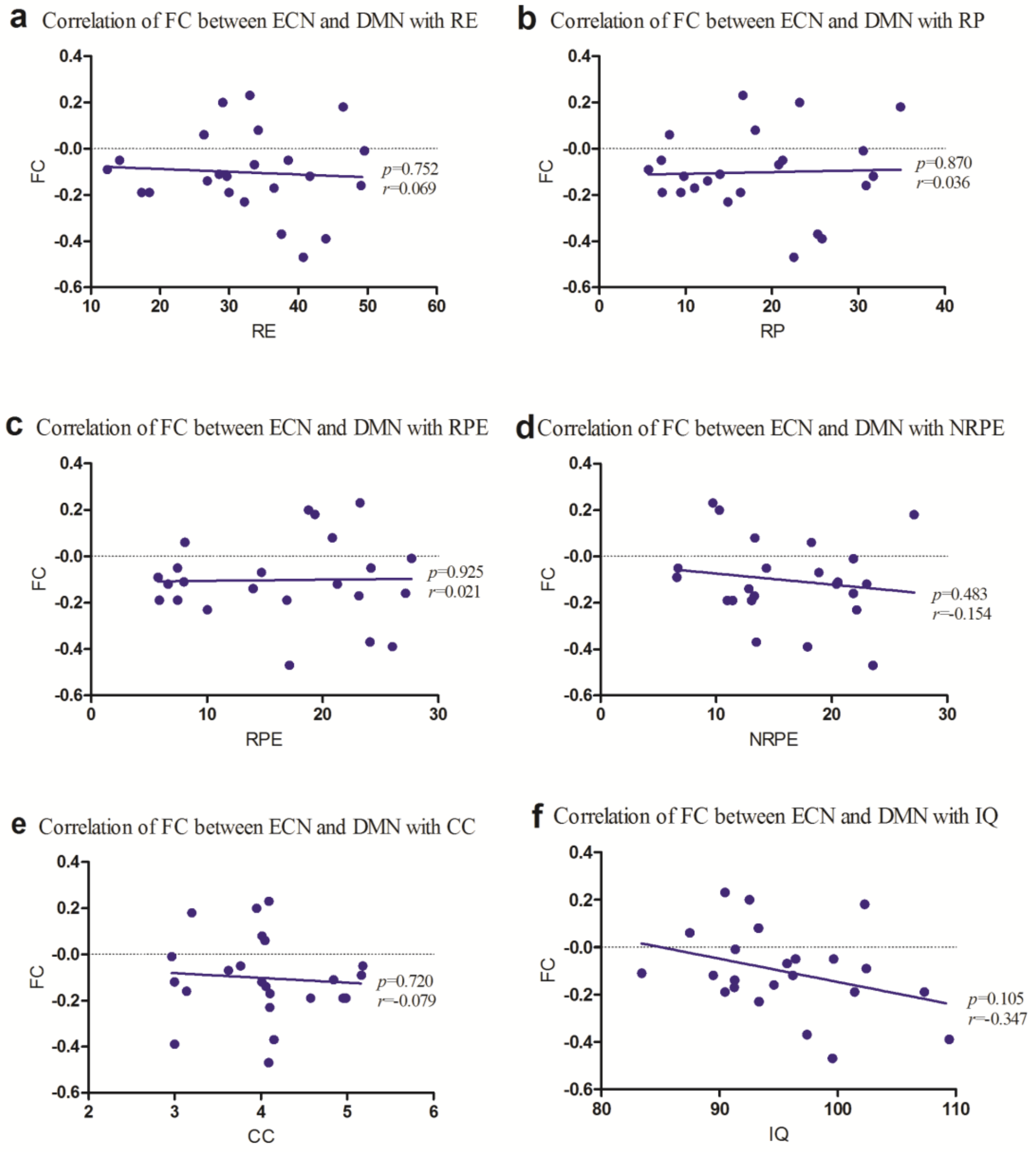

Supplementary Figure 2. Correlations of FC between ECN and DMN with WCST and IQ scores in healthy controls. No significant correlation was found between FC with WCST and IQ scores (p>0.05).

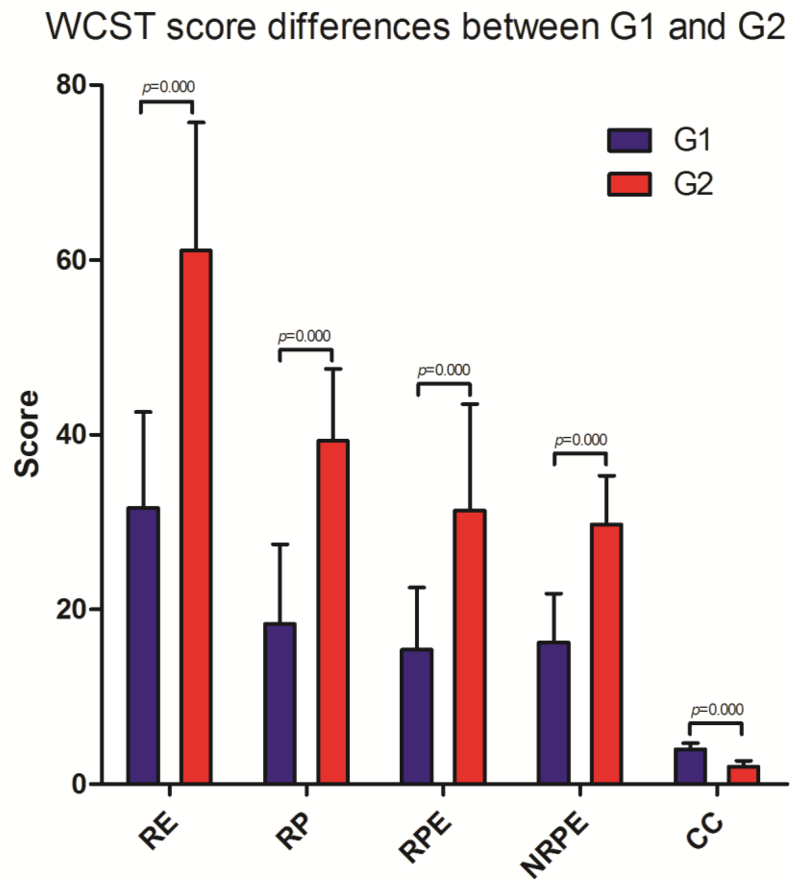

Supplementary Figure 3. Intergroup difference of WCST tests between G1 and G2. Significant differences of WCST scores of RE, RP, RPE, NRPE and CC was found between G1 and G2 (*p*=0.000). CC, categories completed; G1, subgroup 1; G2, subgroup 2; RE, response errors; RP, perseverative responses; RPE, perseverative errors; NRPE, nonperseverative errors.
